# COVID-19: molecular pathophysiology, genetic evolution and prospective therapeutics—a review

**DOI:** 10.1007/s00203-021-02183-z

**Published:** 2021-02-08

**Authors:** C. T. Dhanya Raj, Dinesh Kumar Kandaswamy, Ravi Chandra Sekhara Reddy Danduga, Raju Rajasabapathy, Rathinam Arthur James

**Affiliations:** 1 Department of Marine Science, Bharathidasan University, Tiruchirappalli, Tamilnadu 620024 India; 2 Department of Epidemiology and Public Health, Central University of Tamilnadu, Thiruvarur, Tamil Nadu India; 3 Present Address: School of Optometry and Vision Sciences, Cardiff University, Maindy Road, Cardiff, CF24 4HQ UK; 4 Department of Pharmacology, University College of Pharmaceutical Sciences, Acharya Nagarjuna University, Guntur, Andhra Pradesh India

**Keywords:** COVID-19, Drug repositioning/repurposing, Molecular targets, Remdesivir, Favipiravir, Ribavarin, Dexamethasone

## Abstract

The Covid-19 pandemic is highly contagious and has spread rapidly across the globe. To date there have been no specific treatment options available for this life-threatening disease. During this medical emergency, target-based drug repositioning/repurposing with a continuous monitoring and recording of results is an effective method for the treatment and drug discovery. This review summarizes the recent findings on COVID-19, its genomic organization, molecular evolution through phylogenetic analysis and has recapitulated the drug targets by analyzing the viral molecular machinery as drug targets and repurposing of most frequently used drugs worldwide and their therapeutic applications in COVID-19. Data from solidarity trials have shown that the treatment with Chloroquine, hydroxychloroquine and lopinavir-ritonavir had no effect in reducing the mortality rate and also had adverse side effects. Remdesivir, Favipiravir and Ribavirin might be a safer therapeutic option for COVID-19. Recent clinical trial has revealed that dexamethasone and convalescent plasma treatment can reduce mortality in patients with severe forms of COVID-19.

## Background information

Severe Acute Respiratory Syndrome coronavirus 2 (SARS-CoV-2), previously known as 2019 novel coronavirus (2019-nCoV), is a novel species of *Coronaviridae* family, which causes COVID-19, the ongoing pandemic outbreak across the globe. SARS-CoV-2 was first reported in Wuhan, Hubei Province, China in December 2019 as a novel pathogen causing pneumonia (Zhu et al. 2020). In comparison to other corona viruses (CoV) that caused deadly diseases in the past, SARS-CoV-2 has a stronger transmission capacity thus being highly contagious and causes rapid increase in the infection rate globally (Tang et al. 2020). As of 15^th^ July about 13,070,095 positive cases and around 572,539 deaths were confirmed across the world (WHO 2020). In the past two decades, corona viruses have caused the SARS (Severe Acute Respiratory Syndrome) Outbreak in 2002 and the MERS (Middle East Respiratory Syndrome) in 2015 (Guo et al. 2020). The former has infected about 8000 positive cases with 9.6% fatality rate, while MERS had affected about 2500 cases with 34% fatality rate, respectively (Cascella et al. 2020; Raoult et al. 2020).

### Corona viruses

Corona viruses were first identified in the 1960s and subsequently derived their name due to the presence of crown-like spikes on their surface. Corona viruses are of *Coronaviridae* family classified within the *Nidovirales* order with four genera (α, β, γ, and δ CoV) and may cause mild to severe respiratory or gastrointestinal infections in humans, animals and birds. Human corona viruses (HCoV) belong to α and β- CoV. To date seven known pathogenic HCoV have been documented. Among them, four [229E and NL63 (α-HCoV) and HKU1 and OC43(β-HCoV)] are causative agents of mild coryzal illness similar to the common cold while the remaining three are more virulent zoonotic strains of β-HCoV (SARS-CoV, MERS-CoV, and SARS-CoV-2) that cause severe acute respiratory syndrome (Ye et al. 2020; Schaffer et al. 2010).

### Structure

**Fig. 1:**
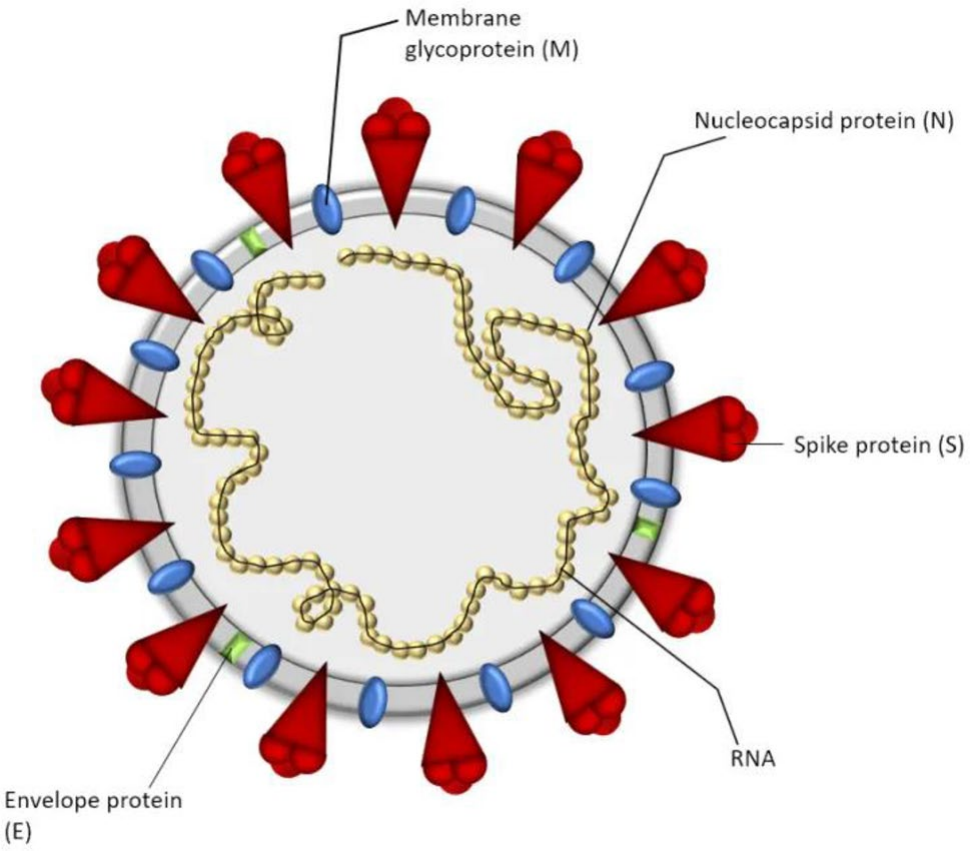
Structure of corona virus, reproduced from Li 2020

In general, HCoV are spherical or pleomorphic enveloped non-segmented positive-sense single-stranded RNA viruses (Fig. 1) that measure about 80–200 nm in diameter with a genome size of 26–30kbp.  The genome consist of untranslated region at both ends (5′-methylated cap and 3′-polyadenylated tail), with two large open reading frames, ORF1a and ORF1b, wherein the non-structural proteins (involved in genome replication such as replicase, proteolytic processing) are encoded within the 5′ region and the structural proteins (envelope protein S (spike), glycoprotein E (envelope) protein, M (membrane) protein and N (nucleocapsid) phosphoprotein) are encoded within the 3′ region. Certain types of corona viruses have additional special structural and accessory proteins such as HE (hemagglutinin-esterase) protein and I (internal) protein. The S protein forms the peplomers on the virion surface which is responsible for the crown-like morphology. It is involved in receptor binding and cell fusion and is a major inducer for neutralizing antibodies. The M protein is the most abundant structural glycoprotein, with three transmembrane domains that define the viral shape. The M protein can interact with all other structural proteins and thus play a key role in viral assembly. E proteins are small structural proteins involved in viral assembly and morphogenesis within the cell. N protein binds with the viral genomic RNA to form a helical capsid structure, thus playing a crucial role in structure, replication and transcription of corona viruses. The HE protein is a non-essential protein which makes smaller spikes on the membrane and acts as hemagglutinin. It binds to the sialic acid present on the spike proteins and enhances the viral entry into the host cell. HE proteins are absent in SARS-CoV (Schaffer et al. 2010; Cecil et al. 2012; Fehr and Perlman 2015).

### Insight into SARS-CoV-2

**Fig. 2:**
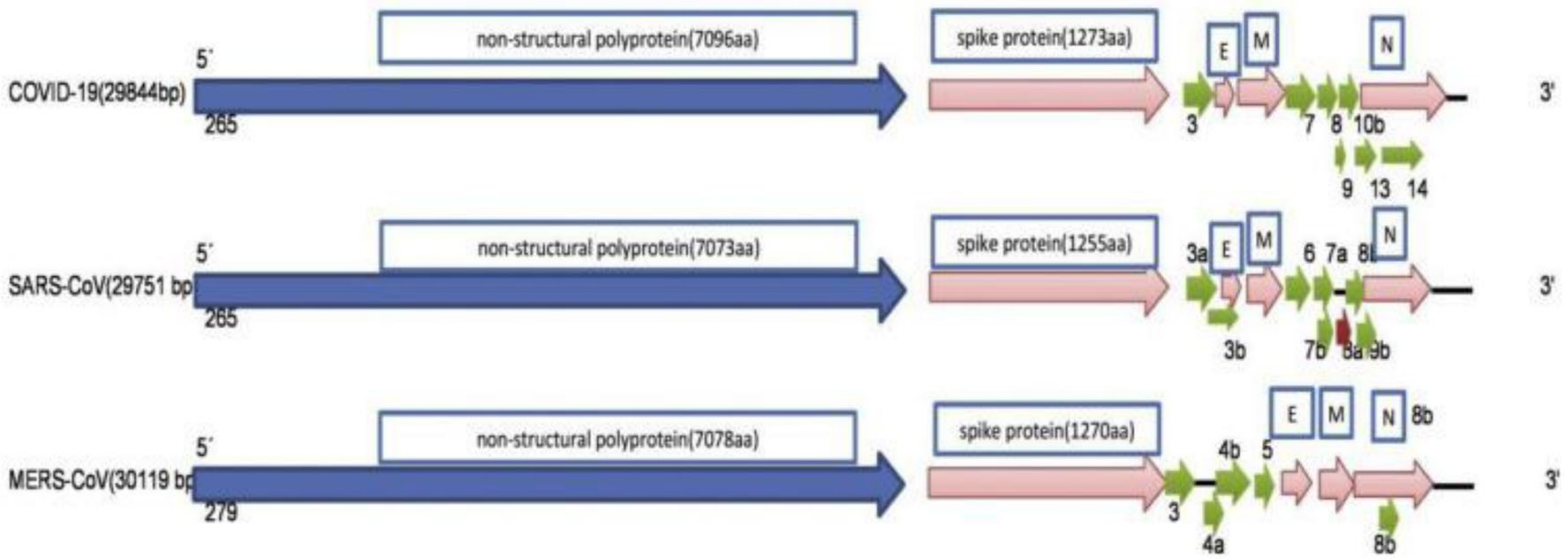
Genome organization of SARS-CoV-2, SARS-CoV, and MERS. Reproduced from Mousavizadeh and Ghasemi 2020

The genome sequences of novel SARS-CoV-2 are 50% similar to MERS-CoV, 79.5% similar to SARS-CoV, and 96.2% similar to a bat corona virus (Bat-CoV) RaTG13. SARS-CoV-2 size measured 60–140 nm diameter with 8–12 nm long spikes (Guo et al. 2020; Yang and Wang 2020). The viral genome has 29,891 nucleotides with a GC content of 38%, encoding 9860 amino acids (Chan et al. 2020; Lu et al. 2020). The genomic encoded proteins are more similar to SARS-CoV which are arranged in the order of 5′—replicase (ORF1a/b)—structural proteins (S- E- M- N)—3′. As a typical corona virus, SARS-CoV-2 also has six ORFs. 5′-replicase genes contain two large open reading frames, ORF1a/b. The frame shift between ORF1a and ORF1b produces two polypeptides: pp1a and pp1ab. These replicase polypeptides are proteolytically processed by virally encoded chymotrypsin-like protease (3CL^pro^) or main protease (M^pro^) and additionally cleaved by papain-like protease (PL^pro^) to generate 16 nonstructural proteins (NSPs 1–16) (Cascella et al. 2020). The other ORFs encode the structural proteins and accessory proteins. Spike (S) protein is divided into S1 and S2 subunits.  The S1 subunit shares 70% homology with SARS-CoV and Bat-CoV, encompasses an N-terminal domain, a receptor binding domain and motif and are involved in receptor binding to the host cell. The S2 Subunit that share 99% homology with SARS-CoV as well as Bat-CoV, comprised of a fusion peptide, a transmembrane domain and a cytoplasmic domain, and are responsible for cell membrane fusion. Like SARS-CoV, the HE protein is absent in SARS-CoV-2 (Chan et al. 2020). Among the 16 non-structural proteins, NSP3 (papain-like protease), NSP5 (3-chymotrypsin-like protease), NSP12 (RNA-dependent RNA polymerase), and NSP13 (helicase) are key enzymes essential for viral transcription and replication process (Fig. 2). Therefore, these four nonstructural proteins and S protein would be attractive sites for antiviral agents (Chan et al. 2020; Lu et al. 2020). Compared to SARS-CoV, the transmembrane helical segments in the ORF1ab NSP2 and NSP3 in SARS-CoV-2 have no evident homologous structures. At 501, 723 and 1010 amino acid positions, glutamine, serine, and proline are present instead of alanine, glycine and isoleucine residues, respectively. The stabilizing mutations located within the endosome‐associated‐protein‐like domain of the NSP2 protein make the virus highly contagious. The destabilizing mutations in the phosphatase domain of the NSP3 proteins suggest differentiation mechanism of SARS-CoV-2 from SARS-CoV (Angeletti et al. 2020).

### Phylogenetic analysis

**Fig. 3:**
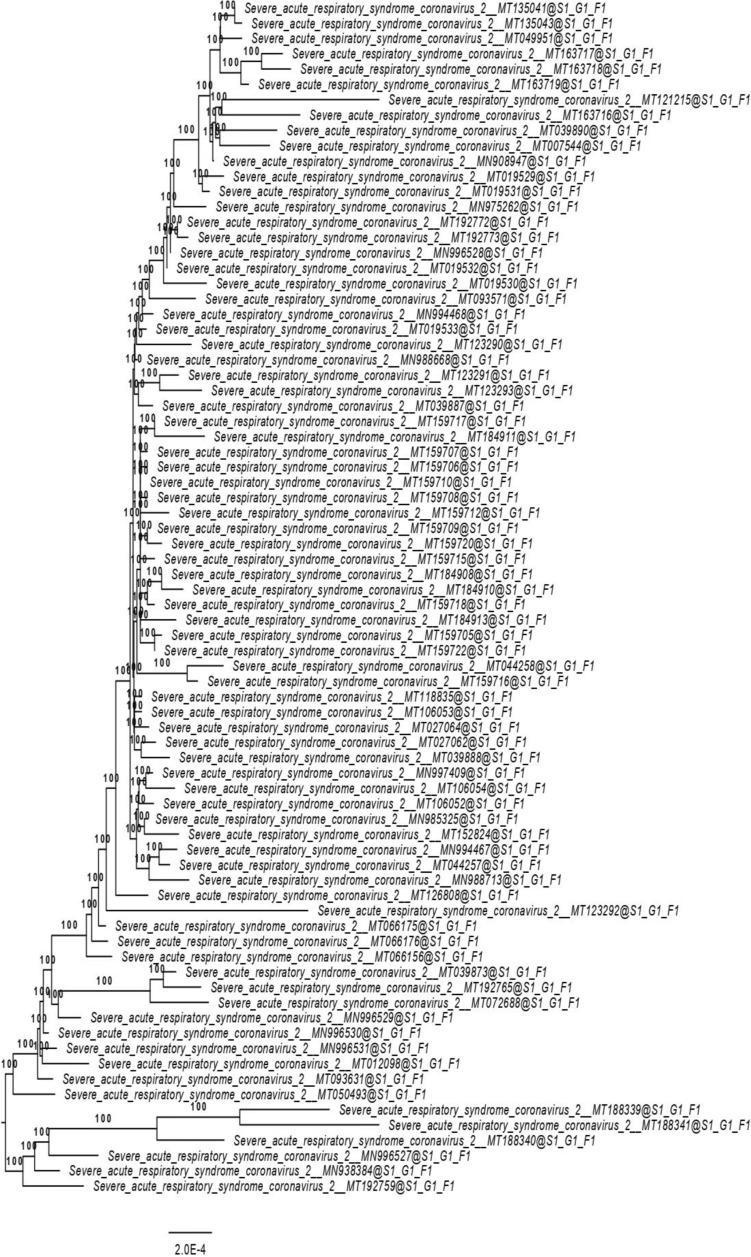
Phylogenomic tree inferred using the formula D6 (GBDP_Trimming_D6_FASTME). The numbers above branches are bootstrap support values from 100 replications

Nearly 15,000 SRA files and 19,000 biosample datasets are available in the GenBank database in which 92 of them are assembled in comparison to SARS-CoV-2 genome. With the available assembled genome, we have tried to perform a phylogenetic analysis to find out the evolutionary distances between each sequence. Pair-wise comparisons of the nucleotide sequences were performed using the Genome-BLAST Distance Phylogeny (GBDP) method (Meier-Kolthoff et al. 2013) under the settings recommended for prokaryotic viruses in VICTOR tool (Meier-Kolthoff and Göker 2017). The resulting inter-genomic distances were used to infer a balanced minimum evolution tree with branch support via FASTME including SPR post processing (Lefort et al. 2015). Trees were rooted at the midpoint (Farris 1972) and visualized with FigTree (Rambaut 2006). Taxon boundaries were estimated with the OPTSIL program (Göker et al. 2009), with recommended clustering thresholds and an F value of 0.5 (Meier-Kolthoff et al. 2014). The phylogenomic tree clearly shows the difference between each genome (Fig. 3). The SARS-CoV-2 genome has just 15 genes, yet it mutates all the time when it spreads. Most of these modifications make very little difference but at times the virus becomes either more/ less contagious (Fell and Davis 2020). Studies on mutations of functional proteins between SARS-CoV-2 and SARS-CoV genomes revealed 996 mutational changes occurred in ORF 1a and 1b amino acid sequences, whereas the comparisons within SARS-CoV-2 genomes showed only 11 changes (Khan et al. 2020).

### Pathogenesis

One could observe some basic structural similarities in all the three zoonotic HCoV; however, the genome sequence and phenotypic arrangements (length of spikes) differ in each case based on genetic and structural analyses. These differences influence their pathogenic and infectious nature. In all the three cases, the initial transmission occurred from animal to human through direct contact and rapid spread occurred among humans through respiratory droplets and direct contact routes. The SARS-CoV-2 viral genomic evolutionary analysis showed that bats are the natural host harboring the virus and the virus might have been transmitted to pangolins, the suspected intermediate host (Zheng 2020), before infecting humans. The SARS-CoV-2 enters the lungs through respiratory tract. The incubation period is 14 days with an average of 3–7 days. In some rare cases the incubation period is reported as long as 24 days (Table 1) (Chan et al. 2020).

**Table 1:** Comparison of SARS, MERS &COVID-19 Outbreaks

Corona virus—epidemic	SARS	MERS	COVID-19
Type of Corona virus	Lineage B βCoV	Lineage C βCoV	Lineage B βCoV
Genome size	27.9 kb	30.1 kb	29.9 kb
Year of outbreak	2002–2003	2012–2013	2019–2020
Place of origin	Guangdong Province, China	Jeddah, Saudi Arabia	Wuhan Province, China
Natural host	Bats	Bats	Bats
Intermittent host	Himalayan palm civet	Camel	Pangolin
Infectious host	Human	Human	Cats and ferrets and poorly in dogs, pigs, chickens, and ducks. Human, cats, ferrets, pigs, dogs, ducks and chickens
Mode of transmission	Direct contact and/or droplet route	Close contact	Direct contact and respiratory droplets
Incubation period	2–11 days	2–13 days	3–7 days
Symptoms	Fever, headache, dry cough, malaise, respiratory distress, diarrhea	Fever, sore throat, cough, myalgia, dyspnea, diarrhea, pneumonia	Fever, dry cough, dyspnea, shortness of breath, headache, myalgia, diarrhea
Infected cases	8096	2500	8,860,331 (as on June 22)
Affected countries	29	20	216
Fatality rates	∼10%	∼34%	∼3.4%

Proteolytic cleavage of the S glycoprotein at S1/S2 and S2′ is the critical step for viral attachment. Protease enzymes such as furin and TMPRSS2 are involved in proteolytic cleavage.

SARS-CoV-2 S glycoprotein has a furin cleavage site which is not present in other HCoV. This enables them to be more infectious and to spread efficiently among humans (Wu et al. 2020a). The furin activation site is targeted by furin protease found in the host cell and cleaves the S protein at S1/S2 site which is essential for cell–cell fusion. The cleaved S1 subunit thus recognizes the ACE2 receptor on the host cell membrane and binds to it. The S protein is activated by the cellular serine protease TMPRSS2 which cleaves at the S2′ site in order to bind to ACE2 receptor. The S2 subunit fuses the host and viral membranes. Viruses enter into the host cell via clathrin-mediated endocytosis and membrane fusion. After entry the virus uncoats and releases nucleocapsid and viral RNA into the cytoplasm, which is then transcribed and translated into structural proteins and two polyproteins. Viral genomic RNA (ssRNA) and subgenomic RNA (mRNA) are synthesized, replicated, and transcribed in the cytoplasm (Mousavizadeh and Ghasemi 2020). Following this, the viral structural proteins are inserted into endoplasmic reticulum and move to ERGIC (endoplasmic reticulum-Golgi intermediate compartment), where assembly of the viral genome, nucleocapsid proteins, and structural proteins occurs. This encapsidated viral genome matures and is multiplied, and released from the cell*.*

### Variation in combating the virus by host immune system—key findings

Lymphocytes (T cells, B cells, and natural killer cells) play an important role in human immune system. During infection, the lymphocyte count increases to fight off the virus. But, in SARS-CoV-2 infection, the lymphocyte count gradually decreases with increasing severity. The mechanism of significant changes of lymphocyte and their correlation with the severity of the disease remains unclear. At early stage of infection peripheral blood lymphocyte count is normal or slightly low; however, lymphopenia is observed in patients. The lymphocyte count of less than 1.5 × 10^9^/L is associated with the severity of COVID-19 (Zhao et al. 2020). B lymphocyte which is responsible for humoral immunity may also decrease in the early stage. This may affect antibody production and thus fail to prevent the viral multiplication (Lin et al. 2020). Another type of lymphocytes which play an important role in viral clearance is T cells (CD8+ and CD4+). Studies have shown that in COVID-19 patients, the total T cell, CD8+ , and CD4+ T cell counts are reduced dramatically, and the surviving T cells are functionally exhausted. This is due to the direct involvement of IL-10 (interleukin-10) which inhibits T cell proliferation and induces T cell exhaustion (Diao et al. 2020).

In some patients, an excessive inflammatory reaction takes place which leads to uncontrolled production of cytokines. These inflammatory responses are known as cytokine storm and cause damage to tissues and organs (Cascella et al. 2020). Another important immunological change in COVID-19 is abnormal increase of IL-6. It is produced by activated monocytes and macrophages which have a pivotal role in cytokine storm and functions through cis or trans signaling pathways. In the cis signal transduction pathway, the IL-6 complex with a transmembrane form of IL-6R (mlL-6R), and membrane bound gp130 activates downstream Janus kinase (JAKs), signal transducer, and activator of transcription 3 (STAT3). This activation leads to pleiotropic effects on immune cells which contribute to cytokine storm.  In trans signal pathway, IL-6 forms complex with soluble form of IL-6R (sIL-6R) and gp130, and activates downstream JAKs and STAT3. The resultant IL-6- sIL-6R- JAK-STATA3 signaling is activated in immune cells leading to cytokine storm (Zhang et al. 2020; Liu et al. 2020a).

### Clinical manifestations

Patients with COVID-19 initially have mild to severe respiratory illness with symptoms of fever, dry cough, sore throat, and fatigue. During the second phase of infection symptoms like headache, dyspnoea, haemoptysis, diarrhoea, lymphopenia, and difficulty in reading and in distinguishing smells have also been reported. The respiratory distress continues to deteriorate in some patients leading to acute respiratory distress syndrome (ARDS) and often requires medical aided respiration through mechanical ventilation by the third week of infection. RNAaemia and acute cardiac injury are other complications (Huang et al. 2020). Based on the currently available information, the severity rate is high in immunocompromised patients, patients with other co-morbid conditions, and elderly adults over 65, with an overall mortality rate of 3.4% (WHO 2020a).

The COVID-19 outbreak can be controlled through early detection of cases by screening and isolation of persons with symptoms. World Health Organization recommends contact and airborne precautions with N95 or higher respirator masks. Use of alcohol-based sanitizers is advisable to protect from COVID-19 (WHO 2020b). Routine cleaning and disinfection by lipid solvents like 75% ether, ethanol, peroxyacetic acid, chloroform, and chlorine-based disinfectants would help to decontaminate the surfaces and thus reduce the spread of COVID-19. SARS-CoV-2 is sensitive to heat and UV rays.

## Potential therapeutics in COVID-19

To date, there is no specific anti-viral drug against COVID-19 which has been a major challenge for researchers trying to save lives and produce biological therapeutics to target SARS-CoV-2. Although little is known about the SARS-CoV-2, based on homology modeling and sequencing, it was found to have an 82% sequence similarity with the SARS-CoV and more than 90% sequence similarity in its essential enzymes (Morse et al. 2020). Therefore, knowing the viral-specific molecular machinery, researchers can effectively deal with SARS-CoV-2. From previous experiences in treating SARS-CoV and MERS-CoV, which may be directly helpful in treating the present outbreak of COVID-19, it is unlikely that any effort made at this point in a panic situation will benefit the patients. So, in this current review, we emphasize the various antiviral drug targets and the pharmacological therapeutic approaches in treating COVID-19.

### Viral molecular machinery as drug targets

Isolated samples from the workers of the Wuhan seafood market revealed the 29.9-kb complete genome of the SARS-CoV-2 (Wu et al. 2020b). The CoV (coronavirus) genome has several open reading frames (ORFs) ranging from 6 to 11 (Song et al. 2019). About two-thirds of the RNA genome is occupied by ORF1a and 1b which translates into polyproteins (PP1a and PP1ab).  The polyproteins further processed into 16 non-structural proteins (NSPs). The NSPs like RNA dependent RNA polymerase (RdRp), 3 C like protease (3CL^pro^), and papain-like protease (PL^pro^) are responsible for viral replication in the host cells by forming replicase transcriptase complex (Snijder et al. 2016). While, the rest of the RNA genome responsible for the formation of mRNA transcripts which produces structural proteins like spike glycoprotein (S), envelope protein (E), membrane protein (M), nucleocapsid protein (N) and other accessory proteins (Cui et al. 2019). So, the major targets for the anti-viral drugs are structural proteins like S along with the NSPs like RdRp, 3CLpro, and PLpro.

### Structural spike glycoprotein (S)

The spike protein has three segments described as ectodomain, transmembrane anchor, and intracellular domain. Clove-shaped spike proteins gather in a trimeric form on the surface of the virus to form the distinctive crown-like appearance, so, it is named as coronavirus. The ectodomain consists of a receptor-binding domain (RBD) S1 and a membrane fusion domain (MFD) S2. These two domains are the main responsible elements in the viral entry into the host cells (Belouzard et al. 2012). SARS-CoV recognizes ACE-2 receptors as receptors for its association with the host cells (Li et al. 2003). Similar to the SARS-CoV, SARS-CoV-2 also recognizes the ACE-2 receptors for its binding to the host cells. Receptor binding domain S1 mediates association with the ACE-2 receptors of the host cells. The spike proteins of both origins have 76% similarity in sequence (Morse et al. 2020). Recent scientific reports revealed that the binding affinity of SARS-CoV-2 to the human ACE-2 receptors is ten times significantly higher than the SARS-CoV. Wrapp et al. also proved that the SARS-CoV RBD specific monoclonal antibodies have not shown the same binding ability to SARS-CoV-2 RBD (Wrapp et al. 2020). Hence, the cross-reactivity of the monoclonal antibodies related to the RBD of coronavirus was proved to be limited.

The importance of spike proteins in ACE-2 receptor binding and fusion of the membrane with the host cells makes it a potent drug target for the development of anti-viral drugs and vaccines. There are many scientific reports stating that the recombinant RBD binds strongly to the human and bat ACE-2 receptors. Further, the recombinant RBD has also prevented the entry of SARS-CoV-2 and SARS-CoV into the human ACE-2 receptor bearing cells, suggesting that the recombinant protein serves as a viral attachment inhibitor against the SARS-CoV. In contrast to the RBD specific monoclonal antibodies, Tai et al. emphasize that the SARS-CoV RBD specific polyclonal antibodies may cross neutralize the SARS-CoV-2 pseudovirus infection, suggesting the development of potent SARS-CoV RBD based vaccine for the prevention of CoV infections (Tai et al. 2020). Research groups have been trying to develop methods for building macrocyclic peptide libraries. Testing of these libraries for potent ligands for the SARS-CoV-2 RBD or to the ACE-2 peptide regions will lead to the development of anti-SARS-CoV-2 macrocyclic peptides.

The SARS-CoV spike protein, S2 domain plays a major role in the fusion of the virus with the host cells, in which the interaction of heptad repeat 1(HR1) and heptad repeat 2 (HR2) results in the formation of six helical bundles (6-HB), thereby promoting the fusion of the virus with the cellular membrane. Several researchers designed and developed fusion inhibitors against SARS-CoV (Liu et al. 2004) and MERS-CoV (Lu et al. 2014). Literature is scarce regarding whether the SARS-CoV-2 also has similar fusion and entry mechanisms to SARS-CoV and MERS-CoV and if it does, whether the S2 domain can also serve as a drug target for the development of fusion inhibitors for SARS-CoV-2. The S2 domain is the most conserved region in the CoV (Buchholz et al. 2004). According to the sequence alignment, both SARS-CoV and SARS-CoV-2 are highly conserved in HR1 and HR2 with 92.6% and 100% overall homology, respectively. Hence, scientific data suggested that SARS-CoV-2-HR2 derived peptide (SARS-CoV-2-HR2P) may act as a fusion inhibitor, indicating that the SARS-CoV-2-HR1 subunit could serve as a potent drug target for the inhibition of virus fusion with the host cells (Xia et al. 2020). Since the S2 domain is highly conserved in both SARS-CoV and SARS-CoV-2, neutralizing antibodies recognizing the RBD in the S protein S2 of the CoV could be a prominent treatment approach having cross-sensitivity (Yu et al. 2020).

In addition, after binding to the host cell ACE-2 receptor, before internalization, the S proteins of the virus are subjected to enzymatic modifications by several enzymes, especially transmembrane serine protease-2 (TMPRSS2). So protein priming is required for the cellular entry of the virus particle, which is a crucial step in the initiation of viral endocytosis (Hoffmann et al. 2020). After endocytosis, the S proteins are subjected to several enzymatic modifications by cathepsin L and B, lysosomal cysteine proteases in the endo-lysosomes in the acidic environment, in order to fuse the membranes and to release the viral RNA into the host cytosol. The lysosomal acidic environment proved to be an essential condition for the activation of several enzymes.  Therefore, increased lysosomal cysteine proteases activity of cathepsin L and B, is highly dependent on lysosomal acidic pH (Ballout et al. 2020). Thus, it is reasonable to speculate that disrupting the binding and fusion of the virus with the host cells are two potential targets for the intervention against SARS-CoV-2.

### Non-structural proteins

#### 3Chymotrypsin-like cysteine protease (3CL^pro^) and papain-like protease (PL^pro^)

Non-structural proteins (NSPs) like 3CL^pro^ and PL^pro^ are responsible for the auto-proteolytic processing of replicase polyproteins encoded within NSP5 and NSP3, respectively. They are responsible for the processing of both structural and NSPs responsible for the replication and generation of new viruses. 3CL^pro^ of SARS-CoV-2 showed 96% homology with the SARS-CoV (Morse et al. 2020).  The 3CL^pro^ processes the C-terminal of the PPs with 11 cleavage sites, while the PL^pro^ Processes the N-terminal of the PPs with 3 cleavage sites (Fehr and Perlman 2015).  The 3CL^pro^ is in the form of dimers capable of generating mature proteins and anchors the transcription / functional replication complex during the replication of viral RNA (Barretto et al. 2005). Hence, small molecules that are targeting 3CL^pro^ of SARS-CoV are expected to be the potential therapeutic agents for the treatment of SARS-CoV-2.

The PL^pro^ of SARS-CoV has only 83% similarity with the SARS-CoV-2 in its sequence (Morse et al. 2020). However, the secondary structures showed the similarity in the active sites of the PL^pro^ may lay the foundation for the development of SARS-CoV PL^pro^ inhibitors that would also be expected to work for the SARS-CoV-2. PL^pro^ also served as a deubiquitinase that is responsible for deubiquitylate host cell proteins, including NF-κB and interferon regulatory factor 3, which leads to immunosuppression in the virus-infected patients (Báez-Santos et al. 2015). So, therapeutic agents that can inhibit the PL^pro^ activity might act as a potent drug molecule against SARS-CoV-2 and its associated immunosuppression in the viral-infected patients.

#### RNA dependent RNA polymerase (RdRp)

After entry into the host cells, the viral genome is released into the cytoplasm. The genome encodes for both structural and non-structural proteins. The RdRp is a product of a highly conserved region in NSP12 of the coronavirus polypeptide, which catalyzes the synthesis of viral RNA and plays a central role in the transcription process with the assistance of other NSP (Fehr and Perlman 2015). Though SARS-CoV and SARS-CoV-2 share 82% homology in their genetic sequence of RdRp, they share a remarkably high degree (96%) protein sequence in both origins (Morse et al. 2020; Gordon et al. 2020a). Apart from NSP12, other NSPs such as NSP13-helicase, NSP14-exoribonuclease, and N-methyl transferase, etc. too rely on the RdRp processivity, fidelity, and template switching, which cannot be ignored in the viral replication process (Fehr and Perlman 2015). Thus, NSP12 processed RdRp may be considered as a primary target for the anti-viral drugs in the treatment of COVID-19.

#### Treatment approaches based on molecular machinery

At present, there are no FDA approved drugs for the treatment of COVID-19. Therefore, identifying a potent antiviral agent to treat the disease is urgent. Currently, treatment is mainly focused on the symptomatic approach and respiratory support. According to WHO, refractory hypoxemia patients are recommended to provide extracorporeal membrane oxygenation despite lung-protective ventilation (WHO 2020c). At this point in a medical emergency, drug repositioning, or drug repurposing from existing drugs is an effective drug discovery process to shorten the time and reduce the cost of drug discovery (Zhou et al. 2020). Although it is essential to develop vaccination or potential therapeutic agents to specifically target the SARS-CoV-2, it is unlikely that any effort made at this moment will benefit the currently infected patients throughout the world.

Based on previous experiences with the SARS-CoV and MERS-CoV, we may learn some treatment approaches by targeting the common molecular machinery of the coronavirus. In the present review, we have focused mainly on the most frequently used anti-viral drugs all over the world and its potential application in COVID-19.

### Viral entry inhibitors

#### Chloroquine and hydroxychloroquine

Chloroquine (CQ) and its derivative hydroxychloroquine (HCQ) are 4-aminoquinolines that have been known as safe and inexpensive drugs for the use of anti-malarial, anti-amoebic, and immunomodulatory activities (Schrezenmeier and Dörner 2020; Liu et al. 2020b). CQ and its analogue HCQ have been reported for their wide-spectrum of anti-viral activity against HIV, CoV, and Influenza viruses (Savarino et al. 2006; Chiang et al. 1996; Yazdany and Kim 2020). In recent *in-vitro* studies CQ and HCQ were found to inhibit SARS-CoV-2 (Liu et al. 2020b; Wang et al. 2020). This previous data and the current reports suggest that the CQ and HCQ may act as a potential pharmacological agent in preventing or treating the COVID-19.

The molecular mechanism in dealing with the CoV has not been fully elucidated and findings from the previous literature have suggested that the CQ and HCQ may act through a series of steps. Both CQ and HCQ are weak bases and they can penetrate the acidic cell organelles like endosomes and lysosomes, in non-protonated form. Once penetrated, the drug molecules convert into a protonated state to accumulate and alter the pH of the acidic cell organelles, which results in endosomal trafficking and prevents viral fusion into the cells. Further, the altered pH interfering with the cleavage of S proteins and glycosylation of cellular receptors of SARS-CoV results in impairment of viral entry into the host cells and prevents subsequent spread of infection (Vincent et al. 2005). The dual activity of anti-inflammatory and anti-viral activity of CQ and HCQ has been proposed to account for their utility in treating or preventing the SARS-CoV-2 and/or its related symptoms.

In vitro studies on SARS-CoV-2 exhibited a better activity of HCQ than the CQ in lowering the EC_50_ values (Wang et al. 2020). In addition, HCQ has fewer concerns about drug–drug interactions and better safety profile and allows higher daily dose in comparison to chloroquine, leading us to think HCQ may act as a promising drug for the treatment of COVID-19 (Marmor et al. 2016; Yao et al. 2020). Data supporting the treatment of COVID-19 with CQ and HCQ were quite scarce. A new brief report from China reported that the treatment with CQ enhanced viral clearance, improved radiological findings, and reduced disease progression (Gao et al. 2020). However, the data of study design and outcomes were not sent for peer review and published which limits the validity of the study. Another open-label nonrandomized trial conducted on 36 participants with COVID-19 reported improved virologic clearance in 6 participants given only HCQ compared to the 16-person control group of participants receive standard supportive care and the respective percentage of virologic clearance was 70% (14/20) Vs 12.5% (2/16). Further, the study also reported that the combination of Azithromycin to HCQ in 6 participants showed superior viral clearance 100% (6/6) in comparison with the HCQ 57% (8/14) monotherapy (Gautret et al. 2020). Despite the promising results, the study has several limitations such as low sample size in the combination therapy, early cessation of 6 participants from the CQ alone treatment group for intolerance towards medication and no safety outcome reported.

The dosing of the CQ and HCQ for the treatment of COVID-19 recommended based on the pharmacokinetic modeling studies and previous literature is 500 mg orally once or twice daily and 400 mg twice daily for one day, followed by twice daily 200 mg, respectively (Sanders et al. 2020). Both the drugs have a large volume of distribution, nearly every organ like eyes, kidney, lungs, and heart, etc., where retention is prolonged (Barlow et al. 2020).

CQ and HCQ are well-tolerated drugs with common adverse effects; gastrointestinal disturbances such as abdominal cramps, nausea, vomiting, and metallic taste. However, there are increasing safety concerns with both the drugs in increasing the QT interval leads to torsade’s de-pointes, drug-induced sudden cardiac deaths, hypoglycemia, neuropsychiatric effects, and retinopathy (Sanders et al. 2020; Barlow et al. 2020; Chen et al. 2006). Utmost care should be taken while prescribing the medication to patients with pre-existing cardiac diseases and those taking concomitant administration of drugs which prolongs the QT interval. Generally, both drugs are proved to be safe in pregnant women. There are contrary reports where both no significant and adverse effects were observed with doses and duration proposed for the SARS-CoV-2 (Sanders et al. 2020; Barlow et al. 2020).

## Viral protease inhibitors

### Lopinavir/ritonavir

Lopinavir along with Ritonavir (LPV/r) is FDA approved oral combination drug for the treatment of HIV. Lopinavir acts as an HIV-1 protease inhibitor and Ritonavir boosts the activity of Lopinavir by inhibiting both HIV-1 Protease and CYP enzymes’ metabolism of Lopinavir and leads to an increase in the bioavailability (Kempf et al. 1997; Sham et al. 1998). Studies have shown that 3-CL^pro^ is a highly conserved region in different types of coronaviruses in terms of its sequence and 3D structure (Xue et al. 2008). Literature reveals that the HIV-1 protease inhibitors, LPV/r, are the potential drug candidates in inhibiting the 3-CL^pro^ (Nukoolkarn et al. 2008). The protease enzymes are essential for the replication of the generation of new viruses (Morse et al. 2020). In accordance with the literature, LPV/r showed in vitro activity against SARS-CoV and MERS-CoV (Chu Study Group 2004; Wilde et al. 2014), which has promoted research interest in assessing the therapeutic potential of the combination for SARS-CoV-2.

Early reports comparing LPV/r along with the Ribavirin and a historical control group treated with Ribavirin alone revealed a decrease in ARDS caused by SARS-CoV but the historic, observational nature of the control arm prevents definitive conclusions (Chu SARS Study Group 2004). Studies on the SARS-CoV-2 in Vero E6 cells against Lopinovir exhibited anti-viral activity and the estimated EC_50_ at 26.63 µM (Choy et al. 2020), whereas the recent randomized, open-label, controlled trial including patients of COVID-19 received LPV/r (400 mg/100 mg) twice daily for 14 days compared with the standard care revealed no significant difference in viral clearance and mortality rate (Cao et al. 2020). The current data prove the limited therapeutic benefit of LPV/r in the treatment of COVID-19. Treatment with LPV/r may result in several gastrointestinal problems like nausea, vomiting sensation, and diarrhea. In addition, the patients in ICU care with pre-existing liver diseases and congenital cardiac disease are led to hepatic decompensation and QT prolongation, respectively (Barlow et al. 2020). These adverse effects of LPV/r may exacerbate the symptoms of COVID-19 (Sanders et al. 2020). Some other anti-retroviral drugs inhibiting protease and integrase were identified as potential drug molecules by observing their enzymatic activity. Darunavir proved its activity against SARS-CoV-2 in in vitro cell models (Sanders et al. 2020; Dong et al. 2020). Further clinical studies are needed for the clinical benefit of antiretroviral drugs in COVID-19 treatment.

## Viral RNA polymerase inhibitors

### Remdesivir and favipiravir

The molecular machinery, RNA-dependent RNA polymerase (RdRp) is a key enzyme responsible for the replication of viral RNA in the host cells. The high sequence conservation of the enzyme between the several CoV promotes the researchers to target the RdRp by developing the new drug molecules or repositioning of drug molecules. The currently used nucleotide analogues might provide the most promising research avenue towards disrupting the viral replication cycle. However, the exonuclease activity of NSP14 has been limiting the application of nucleotide analogues and the inactivation of the proof reading mechanism of NSP14 has been shown to improve the efficacy of the drugs in treating CoV infections. The current review mainly focuses on three drugs: Remdesivir, Favipiravir, and Ribavirin (Morse et al. 2020).

Remdesivir is an adenosine nucleotide analogue, originally developed for the treatment of Ebola and Marburg virus infections. However, it has not been approved by the FDA for any medical condition. It is a monophosphate prodrug that undergoes metabolism in the tissues into an active form C-adenosine nucleotide triphosphate. The active form of the drug inhibits the viral RNA-dependent RNA polymerase which prevents viral replication in the host cells. The drug proved its activity in several in vitro and in vivo animal studies against filoviridae (Ebola), paramyxoviridae (Nipah), and coronaviridae (MERS-CoV, SARS-CoV-1, and 2) (Huang et al. 2020; Lo et al. 2019; Sheahan et al. 2017; Gordon et al. 2020b). Studies revealed that the intact NSP14 proofreading activity of the virus, responsible for the resistance towards the nucleotide analogue, can be overcome by the administration of Remdesivir (Agostini et al. 2018). In another study, it was proved that the administration of Remdesivir and the combination of interferon β had better effect than the LPV/r and interferon β in both in vitro and a mouse model of MERS-CoV (Sheahan et al. 2020). Intravenous administration of Remdesivir improved the clinical condition of COVID-19 patients (Holshue et al. 2020). In another recent clinical study,COVID-19 patients, who underwent compassionate use of Remdesivir, showed clinical improvement in 36 (68%) out of 53 patients (Grein et al. 2020). But the study was not a controlled study and hence further randomized, placebo-controlled trials were required to draw concrete conclusions on its efficacy. The suppressive effect of Remdesivir but not the eradication of SARS-CoV 2 in immunocompromised patients was also documented in one recent study, thus suggesting the need to develop new therapies to enhance the outcome of long-term effect in immunocompromised patients (Helleberg et al. 2020). The ongoing clinical studies were conducted with a loading dose of 200 mg followed by 100 mg intravenously (https://clinicaltrials.gov/ct2/show/NCT04252664), which also demonstrated its linear pharmacokinetics between 3 and 225 mg, without any evidence of liver and kidney toxicity (Kujawski et al. 2020). Patients with COVID-19 reported having gastrointestinal disturbances and elevated levels of aminotransferase after the administration of Remdesivir therapy for 1–5 days (Kujawski et al. 2020). However, the clinical findings did not confirm whether the adverse effect is due to drug administration or SARS-CoV-2 infection. The current data on the adverse effect profile of the Remdesivir are sparse and mandate further clinical trials.

### Favipiravir

Favipiravir is a licensed drug in Japan for the treatment of Influenza, which can be considered as a potential agent for the treatment of RNA-dependent viral infections. It is a purine nucleotide anti-viral prodrug which selectively inhibits the RdRp by metabolizing into an active form favipiravir ribofuranosyl-5B-triphosphate and also induces lethal viral mutagenesis, making it a virucidal drug. It showed RdRp enzyme inhibition of the Influenza virus at low concentrations (IC_50_ of 0.022 µg/mL), whereas it does not affect the human DNA polymerases even at a higher concentration of 100 µg/mL. Favipiravir exhibited a broad spectrum of antiviral activity against RNA viruses (Furuta et al. 2017). Furthermore, a recent study demonstrated that Favipiravir inhibited the SARS-CoV-2 infection in Vero E6 cells with EC_50_ of 61.88 µmol/L (Wang et al. 2020). Recent clinical data from an open-labeled, controlled clinical trial of Favipiravir showed a better therapeutic response in disease progression and viral clearance from the SARS-CoV-2 infected patients (Cai et al. 2020). A recent prospective, randomized, controlled, open-label multicenter clinical trial revealed the administration of Favipiravir (1600 mg twice daily on the first day and continued with 600 mg twice daily for another 9 days) did not lead to significant improvement when compared with Arbidol (200 mg thrice per day for 10 days), whereas it improved the latency to relieve clinical symptoms like pyrexia and cough. In the same study, the Favipiravir treatment group was observed to have elevated levels of serum uric acid, ALT/AST, and psychiatric symptoms (Chen et al. 2020). One of the recent case report studies also suggested the usage of  Favipiravir for severe and critically ill patients in order to abate the progression of pneumonia and cytokine storm (Takahashi et al. 2020).  Favipiravir also demonstrated rapid antiviral response in SARS-CoV-2 infected patients in the pilot stage study of phase II/III clinical trial and enabled viral clearance within 4 days in 62.5% of patients (Ivashchenko et al. 2020). Recent clinical evidences suggest the relative short-term safety and tolerability regarding total adverse events except that some adverse events like less serious gastrointestinal adverse events and increased blood uric acid levels remain a safety concern. As the pandemic spreads throughout the world, this drug received approval for emergency use in several countries like Italy, Japan, Russia, Saudi Arabia, and India, etc., (Agrawal et al. 2020). The current data are not sufficient to recommend the Favipiravir for the treatment of COVID-19, and several clinical trials are ongoing in an effort to confirm its clinical significance. However, the safety concerns like teratogenicity and QT prolongation have not yet been adequately studied. So, more evidence on the long-term effect of the treatment is needed for the safe and effective usage of the drug in the present pandemic situation (Ivashchenko et al. 2020).

### Anti-inflammatory agents

The surge of Cytokines and inflammation induced by immunological responses towards the SARS-CoV-2 culminates in an acute respiratory distress syndrome. Evidence from animal studies also suggests that the inhibition of inflammation was proved to be effective against SARS-CoV and MERS-CoV infected animals in improving the outcome (Zha et al. 2020). Corticosteroids are the potent anti-inflammatory agents typically used for several inflammatory diseases like rheumatoid arthritis, asthma, and also for several acute respiratory viral infections. However, the clinical outcome of corticosteroids was disappointing and even delayed the viral clearance during the outbreak of SARS-CoV and MERS-CoV (Gangopadhyay et al. 2020). A recent study showed that the administration of corticosteroid (dexamethasone) showed a decreased mortality rate in patients receiving mechanical ventilation (Horby et al. 2020).

### Dexamethasone

Dexamethasone is a corticosteroid with anti-inflammatory and immunosuppressant properties and has been used in the treatment of certain cancer, inflammatory disorders, severe influenza, SARS-CoV, MERS-CoV, and community acquired pneumonia. This drug was given to COVID-19 patients in the United Kingdom’s national clinical trial—Randomized Evaluation of COVID-19 Therapy (RECOVERY (https://www.recoverytrial.net/)), and demonstrated positive preliminary results. This is the first drug to be reported to reduce COVID-19 mortality rate. According to the RECOVERY team, treatment with dexamethasone with a dosage of 6 mg per day for up to 10 days reduced the mortality rate by about one-fifth and one-third in COVID-19 patients requiring oxygen and ventilator support, respectively. It also found that there were no risk-benefits for the patients who did not require respiratory assistance (Horby et al. 2020). WHO included the use of dexamethasone and other steroids in the COVID-19 treatment guidelines. However, further research is required to complement the usage of steroids in the development of new therapeutics in combination with other drugs for COVID-19.

### Recent promising therapeutic approaches for COVID-19

New findings show that triple combination therapy of drugs interferon beta-1b, lopinavir–ritonavir, and ribavirin are safe and effective and reduce the risk of viral resistance. This treatment effectively suppresses viral load within an average of 7 days of treatment by lessening the symptoms, thereby improving the patient’s clinical conditions as well as curtailing the duration of hospitalization (Price 2020; Hung et al. 2020). Another promising therapeutic strategy is the passive immunization of COVID-19 patients using convalescent plasma of recovered COVID-19 donors. The neutralizing antibodies (Nabs), immunoglobulin G (IgG) and immunoglobulin M (IgM), present in the plasma, will inhibit viral entry, limiting viral amplification and promoting prompt therapeutic actions (Rojas et al. 2020).

### Convalescent plasma as an alternative treatment

The current treatment approaches for critically ill COVID-19 patients are still under investigation; only limited evidence is available for a battery of antiviral, antibiotic, antimalarial, and anti-inflammatory agents along with the aggressive supportive care. In the current pandemic situation, multiple clinical trials are ongoing for the repurposing of various pharmacotherapeutic agents in order to prove their safety and efficacy against SARS-CoV-2. In the clinical trials, the anti-viral drugs like remdesivir, favipiravir and antimalarial drugs like hydroxychloroquine were reported to have efficacy in reducing the mortality while improving the health condition of COVID-19 patients; however, one should also consider its potential side effects (Duan et al. 2020). Moreover, the corticosteroid treatment for critically ill patients remains controversial, due to its complications and delayed viral clearance. Since there is no specific antiviral or vaccine for the critically ill patients, there is an urgent need for an alternative treatment strategy like the usage of convalescent plasma (Russell et al. 2020; Shang et al. 2020).

## Concluding remarks

As COVID-19 is spreading rapidly throughout the world, it is necessary to re-purpose the existing approved drugs on an emergency basis in order to discover effective drugs for COVID-19. Several medicines have been given to the patients on a case by case basis with due consideration being given to potential risk and benefits. Current data prove that treatment with Chloroquine, hydroxychloroquine, and Lopinavir along with Ritonavir had adverse side effects. Remdesivir, Favipiravir, and Ribavirin might be safer therapeutics for COVID-19. Recent reports claim that the drug dexamethasone can cut mortality in patients with severe forms of COVID-19 by a third. Target-based drug repurposing and continuous monitoring and recording of results will accelerate drug discovery. Until we find a novel drug or vaccine, it is necessary to consider safe measures such as social distancing, wearing high-quality masks, sanitization, thus cutting-off the route of transmission.
